# The association between Post-COVID syndrome and self-stigma among the adult Israeli population during the COVID-19 pandemic

**DOI:** 10.1186/s12889-026-26489-z

**Published:** 2026-02-06

**Authors:** Tom Polovin, Natalya Bilenko, Dvora Frankenthal, Michal Bromberg, Lital Keinan-Boker, Nadav Davidovitch

**Affiliations:** 1 Faculty of Health Sciences, Ben Gurion University of the Negev, Beer-Sheva, Israel; 2 Epidemiology, Biostatistics, and Community Health science, School of Public Health, Faculty of Health Sciences, Ben-Gurion University of the Negev, Beer-Sheva, Israel; 3 Israel Center for Disease Control, Israel Ministry of Health, Tel-Hashomer, Ramat Gan, Israel; 4 School of Public Health, Gray Faculty of Medical and Health Sciences, Tel Aviv University, Tel Aviv, Israel; 5 School of Public Health, Faculty of Welfare and Health Sciences, University of Haifa, Haifa, Israel; 6 Azrieli Faculty of Medicine, Bar-Ilan University, Safed, Israel

**Keywords:** Post-COVID syndrome, Long COVID, Self-stigma, Internalized stigma, Ethnic disparities, Health inequities, Psychosocial outcomes

## Abstract

**Background:**

In the wake of the SARS-CoV-2 pandemic, a novel condition, termed Post-COVID Syndrome (PCS) has emerged, impacting a significant portion of the global population. Concurrently, the global impact of the virus has extended beyond physical health, leading to the emergence of COVID-19 related stigma. This study investigates the link between PCS and self-stigma, shedding light on this underexplored phenomenon.

**Objective:**

To examine whether exposure to PCS is associated with higher levels of COVID-19–related self-stigma and to identify potential risk factors.

**Methods:**

A retrospective observational cohort study among adult Israeli citizens who tested positive for COVID-19 in a polymerase chain reaction (PCR) test at least twelve weeks before the interview, serving as a pivotal criterion for diagnosing PCS based on self-reported symptoms. The study sample was drawn from the Israeli Ministry of Health’s comprehensive COVID-19 database and data were obtained via a computerized telephone questionnaire conducted from June through December 2021. The quantification of self-stigma entailed the utilization of the COVID-19 self-stigma scale. The study examined the adjusted association between exposure to PCS and self-stigma, controlling for socio-economic characteristics.

**Results:**

Seven hundred fifty-nine participants (48.7% male) responded, with a mean age of 47.2 years (SD = 14.9). A quarter (25.2%) of the study population met the criteria for PCS, and 28.1% met the criteria for self-stigma. A significant association was found between PCS and self-stigma, with individuals experiencing PCS reporting higher self-stigma (*p* < 0.001). Multivariable linear and logistic regression analyses confirmed that PCS was significantly associated with self-stigma modeled as both a continuous and a dichotomous outcome (*R*^2^ = 0.09, *p* < 0.001; OR = 2.5, *p* < 0.001, respectively).

**Conclusion:**

This study underscores a significant association between PCS and self-stigma, independently of sociodemographic factors. Given this association, it is imperative to develop comprehensive strategies aimed at stigma reduction among PCS patients. These findings have importance beyond COVID-19, providing lessons for future pandemics and emergencies.

**Supplementary Information:**

The online version contains supplementary material available at 10.1186/s12889-026-26489-z.

## Introduction

The SARS-CoV-2 pandemic has affected a substantial proportion of the global population, with over 778 million confirmed cases reported as of December 12, 2025 [1]. This unprecedented pandemic has highlighted the potential dangers of infectious diseases [2]. The fear and rapid spread of the virus, coupled with an infodemic (the spread of harmful misinformation) [3], have also led to an increase in pandemic-related stigma and discrimination [4, 5].

Stigma, especially public stigma, is an undesirable social process of labeling, stereotyping, and prejudice that results in separation, devaluation, and discrimination [6, 7]. Self-stigma, derived from public stigma, has been defined as the internalized acceptance of stereotypes and prejudice [8].

COVID-19-related stigma poses a significant threat to the lives and well-being of patients, healthcare providers, and COVID-19 convalescents [9]. Instances of stigmatization, including healthcare providers being denied access to public transportation, convalescents being stalked on social media, patients rejected by their family members, relatives, neighbors, and colleagues, have occurred worldwide during the pandemic [9, 10].

A systematic meta-analysis on the prevalence of infectious diseases-related stigma, including COVID-19, found an overall stigma prevalence of 34%, with 38% in patients and 36% in the community [5]. COVID-19-related stigma was also reported by healthcare providers, who experienced it from friends and family members [11].

Some individuals affected by the COVID-19 virus may experience symptoms characterized by the National Institute for Health and Care Excellence (NICE) as “Post-COVID Syndrome” (PCS). Three meta-analyses suggest that 10–60% of individuals infected with SARS-CoV-2 experience PCS [12–14]. This syndrome is characterized by signs and symptoms that develop during or after COVID-19, persist for more than 12 weeks, and are not explained by an alternative diagnosis [15, 16]. A systematic review aimed at understanding the experiences of people living with PCS found that stigma is related to PCS, with individuals describing shame, blame, and fear of being stigmatized by the community for having the syndrome [17]. People with PCS reported anxiety due to disbelief from those around them, including healthcare providers [17, 18]. The lack of knowledge among healthcare professionals has been reported to contribute to patients’ feelings of guilt, shame, and anger, which may be further exacerbated by perceived stigma [18]. Recent evidence further indicates that PCS stigma—both anticipated and internalized—is strongly associated with reduced well-being and increased psychological burden [19].

At the time of writing, Israel had over 4,800,000 diagnosed cases of COVID-19, translating to approximately 500,000 individuals with PCS, assuming a conservative prevalence of ~ 10% [1]. Given the lack of a universally accepted definition and limited public awareness about the syndrome, individuals with PCS may not be consistently recognized as such, complicating diagnosis and care. Additionally, asymptomatic transmission of COVID-19 can also lead to PCS, further complicating diagnosis and treatment [20].

The literature highlights that stigma and COVID-19 susceptibility are both strongly influenced by key social determinants of health such as income, education level, and structural racism [21, 22]. These factors are pivotal in exacerbating health inequalities during the global COVID-19 pandemic. For instance, data published by the Catalan government in Spain indicates that COVID-19 infections occurred six or seven times more frequently in the most economically deprived regions compared to the least deprived [23]. Moreover, a different study found significant overlaps between the COVID-19 pandemic and the HIV epidemic among African Americans. This research reveals that structural factors and healthcare stigma, including residential segregation, structural racism, and concentrated poverty, independently contribute to increased susceptibility to both HIV and COVID-19 [24].

Similarly, a systematic meta-analysis highlighted varying levels of stigma prevalence between participants from low- and middle-income countries compared to those from high-income countries (37% vs. 27%, respectively), although this difference was not statistically significant. A significant trend was observed when comparing individuals with lower education levels to higher education levels (47% vs. 33%, respectively), and this difference was found to be statistically significant [5].

Recent OECD indicators show that Israel has a high relative poverty rate (16.8%) and above-average income inequality (Gini 0.345), ranking among the highest in OECD countries [25]. These structural inequalities are reflected in well-documented socioeconomic gaps both between major population groups—such as persistent Jewish–Arab disparities in income and access to health services [26, 27]—and within Jewish subgroups, including marked differences between secular and religious groups [28].

The present study provides a unique opportunity to examine the prevalence of self-stigma among COVID-19 convalescents and to explore associated risk factors, including socio-economic status (SES). In this study, PCS was conceptualized as the exposure of interest, and self-stigma as the primary outcome. Our aim was to determine whether individuals with PCS report higher levels of self-stigma compared with those without PCS, thereby contributing to a clearer understanding of the psychosocial consequences of COVID-19. 

## Methods

### Study design and setting

Our study is part of a broader investigation led by the Israel Center for Disease Control (ICDC), Israeli Ministry of Health [29]. We conducted a retrospective observational cohort study using a structured telephone questionnaire administered in Hebrew and Arabic between June 15 and December 20, 2021.

### Participants

This study cohort consisted of 759 Israeli citizens aged 21 and older who tested positive by PCR for SARS-CoV-2 between January 01 and March 31, 2021, during Israel’s third wave of COVID-19. Inclusion criteria required that participants had received a positive PCR test at least twelve weeks before the interview, but not more than one year prior [30]. This timeframe is commonly used to define PCS in epidemiological studies based on self-reported symptoms. Participants were excluded if they were not Israeli citizens, lacked a valid telephone number, resided in institutional settings (e.g., nursing homes), were unable to be interviewed (due to language or cognitive barriers), had their COVID-19 status confirmed solely by serological testing, or experienced the most severe acute phase of the disease (i.e., required mechanical ventilation or ECMO support).

### Data sources and measurements

The study sample was drawn from the Israeli Ministry of Health’s comprehensive national COVID-19 database. A fixed stratified sampling approach was used to select participants, accounting for key stratification variables such as time since the index SARS-CoV-2 PCR test, gender, age, and population group (80% Jews and 20% Arabs, reflecting Israel’s demographic distribution). This sampling strategy ensured a representative and unbiased study population. The national database is updated daily and includes information on test dates and types (PCR, antigen, serology), vaccination status, age, sex, test results, and the POINTS neighborhood socioeconomic index. To preserve participant privacy, the research team received only minimal contact details for initial recruitment; all direct identifiers were removed immediately after the interviews, ensuring full de-identification of the analytic dataset. The full questionnaire used in this study is provided in Supplementary *File 1*.

### Sample size calculation and power

The sample size was determined a priori to detect an association between PCS and self-stigma. Based on meta-analyses of infectious diseases, including COVID-19 [5], and studies on chronic pain and other chronic health conditions where internalized stigma is prevalent [31, 32], we estimated a baseline self-stigma prevalence of approximately 30% among individuals with PCS. To achieve 80% statistical power (α = 0.05) to detect a moderate and clinically meaningful effect size (defined as an odds ratio ≥ 2.0 for the association between PCS and self-stigma), the minimum required sample size was 684 participants. The final sample size of 759 was therefore sufficient to achieve the targeted statistical power.

### Outcome measure

#### Self-stigma variable

Self-stigma served as the primary outcome of the study. The “COVID-19 self-stigma scale” consists of six questions designed to capture emotional and cognitive aspects of stigma. Although collectively referred to as a “self-stigma scale”, the items reflect two distinct dimensions: internalized stigma (e.g., shame, guilt, avoidance) and perceived stigma (i.e., expectations of negative reactions from others), and no enacted-stigma items were included. These items were adapted from the comprehensive ICDC questionnaire and capture core domains described in established stigma scales [33–36]. The full set of items is provided in Table 1.

**Table 1:** COVID-19 self-stigma scale questionnaire

	Strongly disagree	Disagree	Agree	Strongly agree
I prefer not to tell others that I had COVID-19.				
People may treat me differently because I had COVID-19.				
People will be afraid to get close to me if they know I had COVID-19.				
Because I had COVID-19, I reduce my contact with others.				
I am embarrassed/ashamed that I had COVID-19.				
I live with guilt that I may have infected others with COVID-19.				

Each of the six items on the “COVID-19 self-stigma scale” was rated using a four-point Likert-type scale with responses ranging from “strongly disagree” to “strongly agree”. The total score on the scale ranged from 0 to 18, with a higher score indicating a greater level of stigma.

Self-stigma was operationalized by two ways: as a continuous and as a dichotomous variable. The latter was defined by identifying the upper quartile of the scale, whereby individuals who scored 7 and above were classified as meeting the criteria for self-stigma.

### Exposure and predictor variables

#### PCS variable

PCS was conceptualized as the main exposure variable. Although the literature provides extensive information on the prevalence and characteristics of PCS symptoms [30, 37], no universally accepted diagnostic framework exists. To address this gap, we developed an 18-item self-reported symptom scale. Item selection was guided by early clinical reports and available guidelines, and was designed to be consistent with the symptom domains outlined in the NICE rapid guidelines for PCS [38]. The scale includes symptoms from three major clinical domains:

1. Respiratory: cough, shortness of breath, chest pain [39, 40].
2. Neurocognitive and sensory: concentration or memory difficulties, headaches, sleep disturbances, loss of smell or taste [41–43].
3. Systemic and general: fatigue or exhaustion, fever, decreased physical fitness, muscle pain, joint pain, and hair loss [44–46].

In addition, the scale captures functional limitations in daily life, including difficulty performing household activities, persistent limitations in daily activities, difficulty performing tasks, challenges in planning or time management, and reduced work pace [45–47].

Each symptom reported as more pronounced at the time of interview than before the COVID-19 infection/PCR test was assigned one point on the PCS scale.

To define PCS, we applied a data-driven distributional criterion aimed at identifying participants with the highest symptom burden. A cutoff of ≥ 5 symptoms (i.e., exceeding the 75th percentile of the scale distribution) was selected; importantly, this threshold was determined based on the symptom-count distribution and not on its association with self-stigma. To ensure robustness, sensitivity analyses using alternative cutoffs (≥ 4 and ≥ 6 symptoms) were conducted. These analyses yielded comparable results in direction and significance, indicating that the association between PCS and self-stigma was not dependent on the specific threshold chosen. This approach ensures that PCS classification captures the subgroup with the most substantial symptom load and enables robust comparisons across groups.

Based on this dichotomous PCS definition (yes/no), participants were classified into PCS and non-PCS groups.

### Covariates and other predictor variables

The following demographic, clinical, and behavioral variables were collected and included as potential confounders in the multivariable analyses: sex, age, district of residence, ethnicity, religious status, number of children, employment status, income, existing chronic conditions, active smoking, and time elapsed between diagnosis and the interview.

### Statistical analysis

Statistical analyses were performed using R software version 4.2.3. Reliability of the PCS symptom scale and the self-stigma scale was assessed using Cronbach’s α. Descriptive statistics (means, medians, standard deviations, variances, ranges and quartiles) were calculated for all study variables according to their measurement level. The distribution of the self-stigma score, the primary outcome, was examined and was approximately normal, allowing the use of parametric tests.

Univariable analyses were conducted to compare characteristics between participants with and without PCS. Categorical variables were compared using χ² tests, while continuous variables were compared using independent-samples t-tests. Associations between continuous variables were examined using Pearson or Spearman, as appropriate.

Multivariable regression models were conducted to estimate the adjusted association between PCS and self-stigma. Linear regression was applied when modeling self-stigma as a continuous outcome, and logistic regression was conducted when modeling self-stigma as a dichotomous variable (upper quartile as cutoff). All models controlled for relevant sociodemographic and clinical covariates.

## Results

### Participant characteristics

Basic demographic and socio-economic characteristics of 759 COVID-19–positive participants are described in Table 2. The sample consisted of 48.7% males with a mean age of 47.2 ± 14.9 years. Around one-third of the participants were from peripheral districts, and 18.2% were from the Arab sector. Most participants (75.4%) identified as religious or traditional. Approximately one-quarter (25.7%) were unemployed, and 58% reported having at least one chronic condition. The overall survey response rate was 50.8%. Of the total sample, 191 participants (25.2%) met the criteria for PCS. Self-stigma was identified in 213 participants (28.1%).

**Table 2:** Baseline characteristics and socio-economic variables of the study population

Variable	Study’s population	PCS		*p*- value
		Yes (score > 4) 191 (25.2%)	No (score ≤ 4) 568 (74.8%)	
Sex, n (%)	759 (100%)	-		< 0.001
Male	370 (48.7%)	56 (29.3%)	314 (55.3%)	
Female	389 (51.3%)	135 (70.7%)	254 (44.7%)	
Age, y (mean ± SD)	47.2 ± 14.9	50.9 ± 13.8	45.7 ± 15.1	< 0.001
District, n (%)	727 (100%) *	-		0.103
Periphery	224 (30.8%)	66 (35.9%)	158 (29.1%)	
Central	503 (69.2%)	118 (64.1%)	385 (70.9%)	
Ethnicity, n (%)	759 (100%)	-		0.034
Arab	138 (18.2%)	45 (23.6%)	93 (16.4%)	
Jewish	621 (81.8%)	146 (76.4%)	475 (83.6%)	
Religious status, n (%)	698 (100%) *	-		0.111
Secular	172 (24.6%)	35 (19.9%)	137 (26.2%)	
Religious	526 (75.4%)	141 (80.1%)	385 (73.8%)	
Number of children (mean ± SD, median)	3 ± 2.6, 3	3.3 ± 2.3, 3	2.9 ± 2.7, 3	0.004
Time between diagnosis and interview (mean ± SD, median)	286.8 ± 27.4, 293	297 ± 21.6, 300	283 ± 28.3, 289	< 0.001
Employment status, n (%)	730 (100%) *	-		0.422
Unemployed	188 (25.7%)	52 (28.3%)	136 (24.9%)	
Employed	542 (74.3%)	132 (71.7%)	410 (75.1%)	
Income, n (%)	582 (100%) *	-		0.993
0–8000 NIS**	241 (41.4%)	61 (41.8%)	180 (41.3%)	
> 8000 NIS	341 (58.6%)	85 (58.2%)	256 (58.7%)	
Existing chronic condition, n (%)	759 (100%)	-		< 0.001
Yes	440 (58%)	145 (75.9%)	295 (51.9%)	
No	319 (42%)	46 (24.1%)	273 (48.1%)	
Active smoking, n (%)	727 (100%) *	-		0.93
Yes	119 (16.4%)	31 (16.8%)	88 (16.2%)	
No	608 (83.6%)	153 (83.2%)	455 (83.8%)	

*PCS *Post-COVID Syndrome, *NIS *New Israeli Shekel

* Missing data not presented

** The median income in Israel was approximately 8,500 NIS per month in 2021

### Reliability of the scales

For the COVID-19 self-stigma scale, the resulting Cronbach’s α coefficient was 0.77, indicating good internal consistency [48]. Similarly, for the PCS scale, the Cronbach’s α coefficient was 0.79.

### Univariable and multivariable analyses of the association between PCS and self stigma

Univariable and multivariable analyses were conducted with self-stigma defined as the primary outcome variable. We first examined the univariable associations between self-stigma and each independent variable, including PCS (the main exposure) and the sociodemographic variables. As shown in Table 3, self-stigma was significantly associated with PCS, as well as with SES variables (e.g., residency and income), ethnicity and age (the latter observed only in the continuous-variable analysis). In separate univariable analyses, PCS was associated with ethnicity, as well as sex, age, number of children, time between diagnosis and interview, and existing chronic conditions. Based on these findings, all variables associated with either PCS or self-stigma at *p* < 0.05 were selected as covariates for inclusion in the multivariable models.

**Table 3:** Comparative rates of self-stigma according to selected variables

Variable	Self-stigma (score ≤ 7)		OR	95% C. I		*p*-value
				lower limit	upper limit	
Sex, n (%)	Male	Female	0.93	0.68	1.27	0.706
	101 (27.3%)	112 (28.8%)				
Age, n (%)	0–60 y	> 60 y	1.33	0.9	1.93	0.172
	161 26.8%	52 32.7%				
PCS, n (%)	Yes	No	2.43	1.72	3.44	**< 0.001**
	81 (42.4%)	132 (23.2%)				
District, n (%)	Periphery	Central	1.83	1.3	2.57	**< 0.01**
	81 (36.2%)	119 (23.7%)				
Ethnicity, n (%)	Arab	Jewish	3.31	2.26	4.86	**< 0.001**
	69 (50%)	144 (23.2%)				
Religious status, n (%)	Secular	Religious	0.71	0.47	1.06	0.124
	39 (22.7%)	153 (29.1%)				
Employment status, n (%)	Unemployed	Employed	1.43	0.99	2.04	0.065
	62 (33%)	139 (25.6%)				
Income, n (%)	0–8000 NIS**	> 8000 NIS	1.55	1.08	2.23	**0.023**
	81 (33.6%)	84 (24.6%)				
Existing chronic condition, n (%)	Yes	No	1.13	0.82	1.56	0.51
	128 (29.1%)	85 (26.6%)				
Active smoking, n (%)	Yes	No	1.37	0.89	2.07	0.18
	39 (32.8%)	160 (26.3%)				

*PCS *Post-COVID Syndrome, *NIS *New Israeli Shekel

** The median income in Israel was approximately 8,500 NIS per month in 2021

*Note*: Boldface values indicate statistical significance (*p*<0.05)

Table 4 presents the results of the multivariable logistic regression analysis in which self-stigma served as the dependent variable and PCS was included as the main independent (exposure) variable, alongside relevant sociodemographic covariates. The presence of PCS was significantly associated with an increased likelihood of self-stigma (OR = 2.41, 95% CI: 1.6–3.6, *p* < 0.001). Furthermore, Arab participants were more likely to experience self-stigma compared with Jewish participants (OR = 3.4, 95% CI: 2.3–5.1, *p* < 0.001).

**Table 4:** Multivariable logistic regression predicting self-stigma (yes/no) from PCS and covariates

Model	Independent variables	OR	CI		*p*	goodness of fit		
			**95%** **Lower Limit**	**95%** **Upper Limit**		**Omnibus**	**-2ll**	Hosmer & Lemeshow
Null							853.4	
Model	**PCS** Yes No (reference)	2.41	1.6	3.6	**< 0.001**	< 0.001	787.02	0.55
	**Ethnicity** Arab Jewish (reference)	3.4	2.3	5.1	**< 0.001**			
	**Sex** Male Female (reference)	0.9	0.6	1.3	0.566			
	**Age**	1.0	1.0	1.0	0.081			
	**Active smoking** Yes No (reference)	1.5	1.0	2.4	0.064			
	**Chronic condition** Yes No (reference)	0.9	0.6	1.3	0.515			

*Note*: Boldface values indicate statistical significance (*p*<0.05)

Model fit improved compared with the null model, with the − 2 log-likelihood decreasing from 853.4 (null model) to 787.0 (final model); omnibus *p* < 0.001. Notably, additional models incorporating all variables associated with self-stigma or PCS in univariable tests did not improve model fit. Following a predefined model-building strategy, only variables associated with either PCS or self-stigma at *p* < 0.05 were included as covariates. Multicollinearity was not a concern (all VIFs < 2). Consequently, the variables time between diagnosis and interview, mean income, employment status, religious status, and residency were excluded from the final model, as they did not materially improve model fit or the PCS–self-stigma estimate.

## Discussion

The present study examined whether PCS, conceptualized as the exposure of interest, is associated with higher levels of COVID-19–related self-stigma, which served as the primary outcome. In a stratified sample of 759 Israeli adults infected with COVID-19 during the third wave of the SARS-CoV-2 pandemic, PCS was found to be significantly associated with increased self-stigma, even after controlling for sociodemographic variables. Specifically, individuals with PCS were more than twice as likely to experience self-stigma than those who did not report PCS symptoms. Beyond this central association, the findings also point to the role of broader sociodemographic factors in shaping self-stigmatizing experiences. Participants’ ethnic background was also strongly associated with self-stigma, with the odds of Arab participants experiencing self-stigma being threefold higher compared to Jewish participants.

### Strengths and limitations

Several limitations of this study should be acknowledged. First, given the observational and cross-sectional nature of the data, causality between PCS and self-stigma cannot be established, and bidirectional influences remain possible. Second, the reliance on self-reported symptoms may introduce recall bias and social desirability bias, providing limited insight into actual symptom fluctuation over time. Third, the absence of a universally accepted diagnostic gold standard for PCS may have led to some misclassification in categorizing participants into PCS and non-PCS groups. Fourth, stigma is a complex, non-pathological construct that is inherently difficult to measure accurately. Finally, the data were collected among individuals infected during the Alpha variant period; therefore, the experience of stigma may have evolved as the pandemic and its variants progressed.

Despite these limitations, the present study offers several significant strengths. To address the inherent challenge of directionality, we drew on existing literature which consistently demonstrates that chronic illness and persistent symptom burden are strongly associated with higher levels of internalized stigma across a range of conditions [31, 49]. While these findings do not establish directionality in our specific sample, they provide strong theoretical support for considering the PCS → self-stigma pathway as plausible. Furthermore, to ensure the validity of our measures, the items used to assess self-stigma were derived from established stigma constructs in the literature, helping to mitigate the challenges of measuring such complex social phenomena. Finally, the study draws on a national COVID-19 testing database and employs a rigorous stratified randomization process to ensure a representative sample of 759 Israeli adults, utilizing laboratory-confirmed PCR tests and a comprehensive symptom inventory to provide a robust examination of these associations.

### Comparison with existing literature

Our findings align with prior research indicating the detrimental effects of COVID-19 infection on a range of health outcomes, encompassing mental health and quality of life [50]. The present study extends this literature by examining PCS, a relatively new and emerging health condition that warrants further research. PCS, like fibromyalgia (FM) and myalgic encephalomyelitis (ME), can be conceptualized as a chronic multisymptom condition characterized by challenges in recognition and diagnosis [51]. These similarities provide a basis for understanding the observed association between PCS and self-stigma, highlighting their shared psychosocial impacts. Our finding that PCS status is significantly associated with higher levels of self-stigma reinforces this shared trajectory. Recent PCS research further supports this notion, showing that stigma constitutes a central component of patients’ lived experience, and reflects broader challenges of symptom invalidation and misattribution in chronic, poorly understood conditions [19]. The direction of this association cannot be determined in a cross-sectional design, but prior studies consistently associate persistent symptom burden with higher internalized stigma [31, 49]. One possible explanation for the study’s findings is a complex interplay between physical health outcomes and psychological well-being. Individuals struggling with persistent PCS, which may not yet be consistently recognized or well understood in clinical practice, may experience heightened emotional distress, leading to self-stigmatizing beliefs about their condition, akin to those observed in similar chronic illnesses. Research on FM has revealed a direct correlation: as symptom severity increases, so does self-stigmatization [52], underscoring the vulnerability to stigma among individuals contending with chronic conditions. Similarly, patients with FM or ME face higher levels of stigma compared to healthy individuals. They also report greater stigma than patients with other chronic diseases, such as rheumatoid arthritis, osteoarthritis, and multiple sclerosis [49, 52], suggesting parallel dynamics may apply to individuals with PCS, a similar multisymptom clinical syndrome [53]. In a separate investigation exploring loneliness among patients with ME, results highlighted how prolonged health challenges accompanied by the stigma of a medically contested illness exacerbate feelings of isolation and loneliness. This isolation, in turn, intensifies self-stigma and perceived stigma associated with the condition [54]. Participants in these studies faced challenges due to stigma, influenced by uncertain medical understanding and skepticism among healthcare professionals [55], mirroring the difficulties seen with PCS as a newly identified condition characterized by diagnostic complexities.

Moreover, PCS is inherently linked to COVID-19; therefore, part of the stigma surrounding PCS may also reflect broader pandemic-related stigma attached to acute COVID-19 illness.

In addition, although ethnicity was not the primary focus of this study, the strong and independent association observed between Arab identity and higher levels of self-stigma warrants contextual consideration. This finding aligns with prior research demonstrating that stigma levels across a range of health conditions—including infectious diseases [5], such as HIV [56], obesity [57], and other health conditions—are shaped by broader social determinants, including structural disadvantage, differential access to healthcare, and historical patterns of discrimination [8, 58].

In the Israeli context, longstanding socioeconomic and health-service inequities between Jewish and Arab populations [26, 27] may contribute to heightened vulnerability to internalized and perceived stigma. This vulnerability may be further amplified by cultural factors; recent qualitative studies on long-COVID in minority groups suggest that strong collective values can intensify self-stigma when individuals feel unable to fulfill familial obligations [59]. Thus, while ethnicity was not a hypothesized driver of self-stigma in this study, the persistence of this association after adjustment suggests that social context may play a meaningful role in shaping stigma experiences, complementing the central finding regarding PCS.

### Implications for research and/or practice

These findings have important implications for both clinical practice and future research. The pervasiveness of pandemic-related and chronic illness-related stigma highlighted in this study underscores its substantial impact on individuals’ well-being. Given the strong association observed between PCS and self-stigma, it is essential to develop comprehensive stigma-reduction strategies. Critically, the significantly higher levels of self-stigma observed among Arab participants in our study highlight that such strategies must not be “one-size-fits-all” but rather culturally and linguistically tailored [60, 61].

Such strategies should encompass a range of interventions, including enhancing education and implementing contact-based interventions (i.e., structured interactions between the public and individuals with lived experience), with a particular emphasis on patient-centered programs [62]. In the context of minority groups, these programs should be integrated within community-based frameworks and involve local leadership to address culturally specific perceptions and social barriers that may exacerbate internalized stigma [63]. Consistent with recent work on PCS-related stigma, interventions that foster self-compassion and reduce self-coldness may further buffer the negative impact of internalized stigma on subjective well-being and psychological flourishing [19]. By addressing stigma-related barriers through a culturally sensitive lens [64], healthcare systems can better support individuals with PCS and improve access, acceptance, and engagement with care.

From a research perspective, there is a clear need for further investigation into the underlying mechanisms driving self-stigmatization in PCS and related chronic conditions, with a specific focus on how cultural and sociodemographic backgrounds influence these processes. Qualitative studies, in particular, can offer deeper insight into patients’ lived experiences, perceptions, and coping strategies, within diverse cultural contexts, thereby informing the development of more inclusive and effective stigma-reduction interventions.

## Supplementary Information

Supplementary Material 1.

## Data Availability

Data are available from the authors upon reasonable request and with permission ofthe ICDC.

## Abbreviations

PCS: Post–COVID Syndrome
PCR: Polymerase chain reaction
SES: Socio–economic status
ICDC: Israel Center for Disease Control
NIS: New Israeli Shekel
FM: Fibromyalgia
ME: Myalgic encephalomyelitis
