# Supplementary material for: The association between Post-COVID syndrome and self-stigma among the adult Israeli population during the COVID-19 pandemic
Source: BMC Public Health. 2026 Feb 6;26:833. doi: 10.1186/s12889-026-26489-z (PMC12973779; doi:10.1186/s12889-026-26489-z)
Supplement: Supplementary file 1 — Supplementary Material 1. [file 12889_2026_26489_MOESM1_ESM.docx]

**Questionnaire for COVID-19 Recovered Patients**

**Israel Center for Disease Control, Ministry of Health, State of Israel**

**Introduction:** Hello, my name is _______ and I am calling on behalf of the Ministry of Health. Have I reached the phone number _________? Is your name ______________? The Ministry of Health is conducting a study to examine the long-term effects of COVID-19 in a sample of people aged 21 and over who were ill, or who were tested but were not ill with COVID-19. The questions will cover topics regarding health, mental state, and quality of life during the COVID-19 pandemic. The questionnaire takes about 15 minutes and is very important for determining health policy in Israel.

- All information you provide is confidential and will be kept anonymously for research purposes only.
- There is no obligation to answer all questions, and you may stop the interview at any time.
- Would you be willing to answer a few questions? **Yes / No**

*(If the answer was "No": Would you agree to answer if I call at another time? Yes / No)*

**Interviewer:**

- Name of interviewer: _________
- Date: __________

**Age and Sex:**

**Q1:** In what year were you born? ___________ (Your age is: ___________)

*(Interviewer: Do not ask - mark the sex of the interviewee)*

- Male
- Female

**General COVID-19 Status:**

1. Have you been diagnosed as a COVID-19 patient in the past?

- Yes
- No
- Refuse
- Don't know

1. When were you diagnosed as a COVID-19 patient?

- In the last month to 3 months
- In the last 4-6 months
- In the last 7-12 months
- Between the last year and a year and a half
- Between the last year and a half and two years

1. As of today, are you physically as active as you were before you became ill?

- More active
- Similarly active
- Less active
- Much less active

1. As of today, are you functioning cognitively (e.g., memory, concentration) as you did before you became ill?

- Functioning better
- Functioning similarly
- Functioning slightly worse
- Functioning much worse

1. As of today, are you socially active as you were before you became ill?

- More active
- Similarly active
- Less active
- Much less active

**Symptoms (Last two weeks):**

1. In the last two weeks, have you suffered from fatigue or exhaustion?

- Yes / No
  - If "Yes": To what extent compared to your condition before you became ill? (More than before / Similar / Less)

1. In the last two weeks, have you suffered from sleep disturbances?

- Yes / No
  - If "Yes": To what extent compared to your condition before you became ill? (More than before / Similar / Less)

1. In the last two weeks, have you suffered from difficulties in concentration or memory?

- Yes / No
  - If "Yes": To what extent compared to your condition before you became ill? (More than before / Similar / Less)

1. In the last two weeks, have you suffered from a decrease in physical fitness?

- Yes / No
  - If "Yes": To what extent compared to your condition before you became ill? (More than before / Similar / Less)

1. In the last two weeks, have you suffered from heart palpitations?

- Yes / No
  - If "Yes": To what extent compared to your condition before you became ill? (More than before / Similar / Less)

1. In the last two weeks, have you suffered from shortness of breath at rest or during exertion?

- Yes / No
  - To what extent do you suffer from shortness of breath compared to your condition before you became ill? (More than before / Similar / Less)

1. In the last two weeks, have you suffered from a cough?

- Yes / No
  - If "Yes": To what extent compared to your condition before you became ill? (More than before / Similar / Less)

1. In the last two weeks, have you suffered from muscle pain?

- Yes / No
  - If "Yes": To what extent compared to your condition before you became ill? (More than before / Similar / Less)

1. In the last two weeks, have you suffered from joint pain?

- Yes / No
  - If "Yes": To what extent compared to your condition before you became ill? (More than before / Similar / Less)

1. In the last two weeks, have you suffered from headaches?

- Yes / No
  - If "Yes": To what extent compared to your condition before you became ill? (More than before / Similar / Less)

1. In the last two weeks, have you suffered from chest pain?

- Yes / No
  - If "Yes": To what extent compared to your condition before you became ill? (More than before / Similar / Less)

1. In the last two weeks, have you suffered from hair loss?

- Yes / No
  - If "Yes": To what extent compared to your condition before you became ill? (More than before / Similar / Less)

**General Condition & Functioning:**

1. How would you generally define your condition from the time you were notified of your recovery from COVID-19 until today?

- Stable
- Fluctuating (ups and downs)
- Improving
- Worsening

1. Since you became ill with COVID-19, do you have difficulty performing tasks that used to be automatic, difficulty planning or managing time, or a work pace slower than usual?

- Yes / No

1. Since you became ill with COVID-19, do you have a persistent limitation that makes it difficult for you to perform daily activities such as moving from place to place, eating, dressing, etc.?

- Yes / No

1. Since you became ill with COVID-19, do you have difficulty performing household activities? (e.g., cleaning, shopping, running errands outside the home, etc.)

- Yes / No

**Symptoms During/After Illness (Retrospective):** *The following questions refer to symptoms that may have appeared during the COVID-19 illness and/or after you recovered (Recovery means receiving medical confirmation).*

1. During the COVID-19 illness or after receiving medical confirmation of recovery, did you suffer from **shortness of breath**?

- Yes / No
  - If "Yes": When? (Mark all that apply: During illness / After recovery / Until today)

1. Did you suffer from a **cough**?

- Yes / No
  - If "Yes": When? (Mark all that apply: During illness / After recovery / Until today)

1. Did you suffer from **headaches**?

- Yes / No
  - If "Yes": When? (Mark all that apply: During illness / After recovery / Until today)

1. Did you suffer from **muscle pain**?

- Yes / No
  - If "Yes": When? (Mark all that apply: During illness / After recovery / Until today)

1. Did you suffer from a sense of **loss of smell and taste**?

- Yes / No
  - If "Yes": When? (Mark all that apply: During illness / After recovery / Until today)

1. Did you suffer from **abdominal pain or diarrhea**?

- Yes / No
  - If "Yes": When? (Mark all that apply: During illness / After recovery / Until today)

1. Did you suffer from **fever**?

- Yes / No
  - If "Yes": When? (Mark all that apply: During illness / After recovery / Until today)

**Chronic Morbidity - BEFORE COVID-19:**

1. Before you became ill with COVID-19, did a doctor ever diagnose you with the following conditions? *(Interviewer: Read all listed diagnoses)*

- Lung diseases (If yes: Is it Asthma?)
- Hypertension (High blood pressure)
- High cholesterol and/or triglycerides
- Heart diseases (If yes: Heart attack / Angina / Heart failure)
- Stroke / Cerebrovascular accident
- Hypercoagulability
- Diabetes (Not gestational diabetes)
- Cancer or malignant tumor (including Leukemia or Lymphoma)
- Anxiety disorder
- Depressive disorder
- Arthritis or Autoimmune disease
- Nutritional deficiencies (Iron, B12, or Vitamin D deficiency) -> (If yes: Anemia / Vitamin D deficiency)
- Migraine
- Liver disease
- Sexual dysfunction
- Neurological disease (e.g., Parkinson's, Multiple Sclerosis, or other)

*For each "Yes": Did you receive medication for the disease in the 12 months prior to COVID-19? (Yes / No / Don't know)*

- 1. Are there any diseases/conditions I did not mention that were diagnosed before you became ill with COVID-19?
- Yes / No (If "Yes": What are they? _________________)

1. Were you hospitalized for at least one night in the year preceding the COVID-19 illness?

- Yes / No
  - How many times were you hospitalized? _____________________

**Chronic Morbidity - AFTER COVID-19:**

1. Since you became ill with COVID-19, has a doctor diagnosed you with **new** diseases/conditions that you did not suffer from before?

- Yes / No
  - If "Yes", which new diseases/conditions from those I read earlier? *(Interviewer: Mark in the table what the interviewee says and ask about medication treatment)*

**Self-stigma Scale:**

1. Here are a number of statements. What is your opinion regarding each statement? *(Scale: Strongly Agree | Agree | Disagree | Strongly Disagree)*

- I prefer not to tell others that I had COVID-19.
- People may treat me differently because I had COVID-19.
- People will be afraid to get close to me if they know I had COVID-19.
- Because I had COVID-19, I reduce my contact with others.
- I am embarrassed/ashamed that I had COVID-19.
- I live with guilt that I may have infected others with COVID-19.

**Social & Lifestyle:**

1. In the last month, to what extent did your physical health or emotional problems interfere with your social activities (such as visiting friends or relatives)?

- All the time
- Most of the time
- Some of the time
- A little of the time
- Not at all

1. **Smoking:** Do you smoke cigarettes (including rolled cigarettes)?

- Yes, currently smoke
- No, I stopped smoking since the COVID-19 illness
- No, I stopped smoking in the past
- No, and I never smoked in the past

1. Before the COVID-19 illness, did you engage in regular physical activity in your free time (e.g., running, muscle strengthening, walking for sport, Pilates, soccer, folk dancing, dance, yoga, ball games, etc.)?

- Yes
- No
- Refuse
- Don't know

1. Since you became ill with COVID-19, has there been a change in your physical activity habits (frequency, effort)?

- Yes, I do more
- Yes, I do less
- The same
- I stopped doing physical activity

**Demographics:**

1. In which locality do you live? _________________
2. **Nationality/Religion:** Are you:

- Jewish
- Arab – Christian
- Arab – Muslim
- Bedouin
- Christian (non-Arab)
- Druze
- Circassian
- Armenian
- Arab (no further details)
- Refuse / Don't know / Other: ___________________________

1. **Self-definition:** Do you define yourself as:

- Secular
- Traditional
- Religious
- Ultra-Orthodox (Haredi)
- Refuse / Don't know

1. **Marital Status:**

- Married or living with a partner
- Divorced or separated / Living separately
- Widowed
- Single
- Refuse / Don't know

1. Do you have children?

- Yes / No (Refuse / Don't know)
  - How many children? ____________________

1. **Employment:** How do you primarily define yourself?

- Employee
- Self-employed
- Unemployed
- Student
- Pensioner / Pensioner who works
- Homemaker
- Yeshiva student
- Soldier in mandatory service
- Not working due to disability
- Kibbutz member
- Refuse / Don't know / Other: ________________

1. **Education:** How many years have you studied in total, including university or vocational studies? __________ (excluding unfinished academic years).
2. **Income:** Before the COVID-19 pandemic, what was the average net monthly household income? *(Note: Net income is approx. 15,427 NIS per month according to CBS 2016)*

- Below 4,000 NIS
- Between 4,001 and 8,000 NIS
- Between 8,001 and 12,000 NIS
- Between 12,001 and 17,000 NIS
- Between 17,001 and 22,000 NIS
- Above 22,000 NIS
- Refuse / Don't know

1. May we contact you again during the coming year for a questionnaire regarding COVID-19?

- Yes / No / Refuse / Don't know

**Comments:** ______________________________________________________

**Thank you very much for your cooperation.**
